# Bronchial stump aspergillosis after lobectomy for lung cancer as an unusual cause of false positive fluorodeoxyglucose positron emission tomography and computed tomography: a case report

**DOI:** 10.1186/1752-1947-5-72

**Published:** 2011-02-22

**Authors:** Ignasi Garcia-Olivé, Felipe Andreo, Òria Rosiñol, Jose Sanz-Santos, Albert Font, Eduard Monsó

**Affiliations:** 1 Pulmonology Department, Hospital Universitari Germans Trias i Pujol, Carretera del Canyet s/n 08916 Badalona, Barcelona, Spain; 2 Pathology Department, Hospital Universitari Germans Trias i Pujol, Badalona, Barcelona, Spain; 3 Oncology Department, Hospital Universitari Germans Trias i Pujol, Badalona, Barcelona, Spain; 4 Pulmonology Department, Corporació Sanitària Parc Taulí. Sabadell, Barcelona, Spain

## Abstract

**Introduction:**

Bronchial stump aspergillosis is a rare entity characterized by cough and hemoptysis.

**Case presentation:**

We report the case of a 58-year-old Caucasian woman who developed bronchial stump aspergillosis two years after a left upper lobe resection for lung cancer. Bronchial stump aspergillosis was diagnosed as a result of a focus of increased fluorodeoxyglucose activity in a follow-up positron emission tomography and computed tomography scan. She was treated with oral antifungal therapy and presented with good evolution after three months of treatment.

**Conclusion:**

Bronchial stump aspergillosis is an unusual complication after pulmonary resection. Clinicians should be aware of it when a local recurrence of cancer around the bronchial stump is suspected based on a positive positron emission tomography and computed tomography finding.

## Introduction

*Aspergillus *species colonization of an endobronchial suture, with the appearance of reacting bronchial granulation tissue, is a rare event that has been associated with the use of silk for sutures [1]. Disease usually appears some months after surgery, generally with cough and hemoptysis [2,3].

We report the case of a woman with a history of lung cancer treated by left upper lobe resection who was diagnosed with bronchial stump aspergillosis (BSA) after increased fluorodeoxyglucose (FDG) activity in a positron emission tomography and a computed tomography (PET-CT) scan initially led to a suspicion of tumor recurrence. Both the PET-CT and bronchoscopic abnormalities decreased after antifungal therapy.

## Case presentation

A solitary pulmonary nodule on the left upper lobe was detected as an incidental finding in a 58-year-old Caucasian woman who was a former smoker with a cumulative exposure of 50 pack-years. She had no other significant history and there was no reason to suspect that she could be immunocompromised. Lung cancer was suspected and an upper left lobe resection was performed two months later and the stumps were sutured with synthetic thread. The pathologic diagnosis was small cell lung carcinoma.

**Figure 1:**
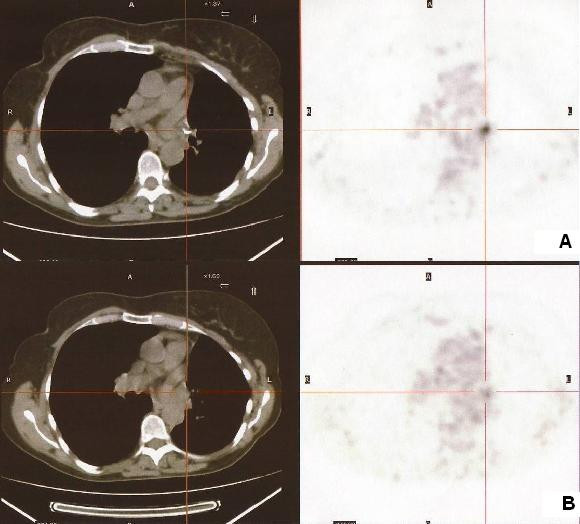
**Fluorodeoxyglucose positron emission tomography and computed tomography scans before (A) and after (B) antifungal therapy**.

**Figure 2:**
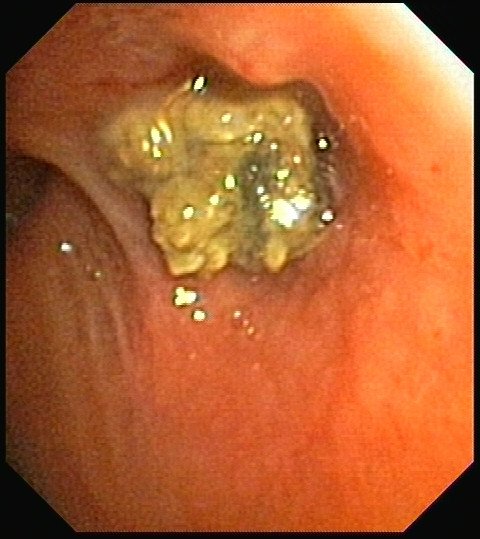
**Necrotic lesion at the left upper lobe bronchial stump as identified at bronchoscopy**.

**Figure 3:**
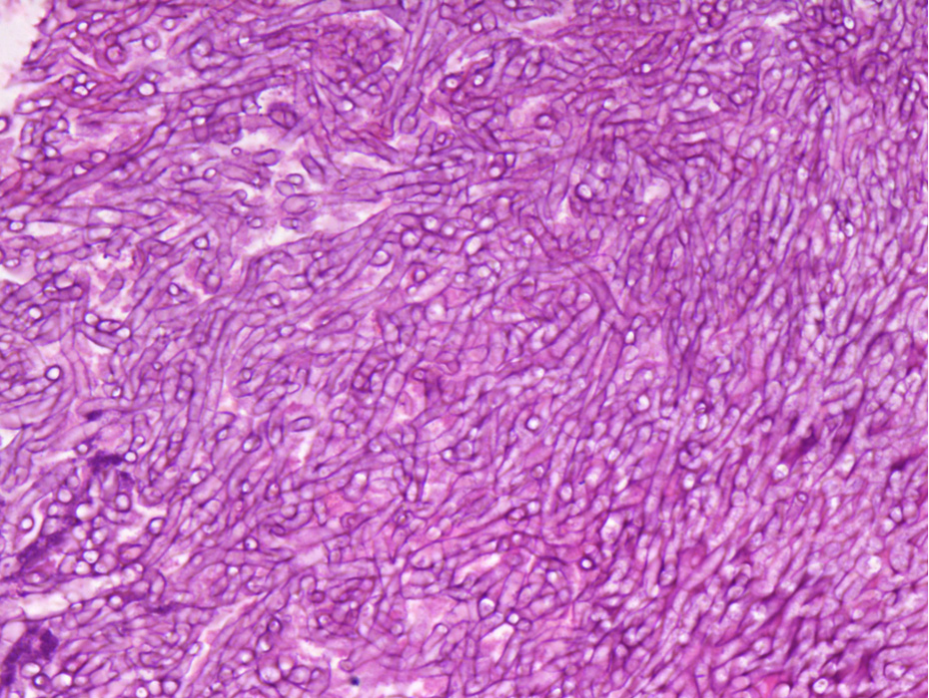
**Chronic inflammation and granulation tissue and numerous hyphae with the appearance of *Aspergillus *species**.

Postoperative chemotherapy and whole-brain radiation therapy were administered and the clinical course was uneventful for two years until a slight rise in serum carcinoembryonic antigen (CEA) and CA 125 levels was detected. A PET-CT scan revealed two foci of increased FDG activity adjacent to left hilar lymph nodes (maximum standardized uptake values [SUVmax] of 10.3 and 8.3, respectively; Figure 1A) and local recurrence was suspected. A necrotic lesion at the left upper lobe bronchial stump was identified at bronchoscopy (Figure 2) and an endobronchial biopsy was obtained. Pathologic examination revealed chronic inflammation and granulation tissue and numerous hyphae with the appearance of *Aspergillus *species (Figure 3) but no sign of malignancy. Cultures were positive for *Aspergillus fumigatus *and antifungal therapy with itraconazole was started and continued for three months. Improvement was remarkable both on a follow-up PET-CT performed two months later (SUVmax of 4.3 and 3.2, respectively; Figure 1B) and a subsequent bronchoscopy, during which fragments of suture were recovered along with biopsy material. Cultures of the material obtained after this bronchoscopy were negative. The patient was followed up once a month for one year after treatment was stopped and there were no signs of recurrence of either the infection or the cancer.

## Discussion

*Aspergillus *species are ubiquitous soil-dwelling microorganisms found in organic debris, dust, compost, foods, spices and rotting plants. Colonization is usually as a result of the inhalation of airborne spores from an inanimate reservoir and, if spores reach the peripheral lung, a variety of clinical syndromes may develop [4]. Although a history of previous lung resection has been described as a risk factor for infection by this fungus [4], few cases of BSA have been reported [1-3] since its description in 1969 as a specific clinical entity [1]. Only five additional cases have been described [2,3,5] since the publication of a series of 17 cases by Noppen and colleagues [2] in 1995. The reported cases have usually been in immunocompetent patients and are associated with the use of silk sutures [1]; a suture made with nylon thread was used in only one case [5]. Aspergillosis often appears with cough and hemoptysis [2,3] but may also be an incidental finding [2].

PET-CT scans are used to distinguish metabolically active from active lesions because of the biochemical differences, especially in staging cancer [6]. However, many positive findings have been reported in nonmalignant diseases such as tuberculosis, other infectious and granulomatous diseases and even benign schwannoma [7,8], not only for the diagnosis but also for monitoring treatment efficacy [9]. The present report of BSA is the first in which the diagnosis came after observing increased FDG activity on a PET-CT scan in an asymptomatic patient. This report suggests that this infection should be included in the range of diagnostic possibilities to consider when follow-up PET-CT scans are positive after lung resection.

Although it is widely suggested that the removal of persisting sutures is the treatment of choice for BSA [1-3,5], good results have also been reported with the use of oral itraconazole as an alternative when removal was not feasible [2]. Given the fact that no trace of suture was found during the initial bronchoscopy procedure, we prescribed antifungal therapy and the response was good.

## Conclusion

Although BSA is rare, clinicians should be aware of this unusual complication after pulmonary resection when local recurrence of cancer around the bronchial stump is suspected based on a positive PET-CT finding. PET-CT can be a useful tool in the monitoring of treatment efficacy in patients with this condition.

## Abbreviations

BSA: bronchial stump aspergillosis; FDG: fluorodeoxyglucose; PET-CT: positron emission tomography and computed tomography; SUVmax: standardized uptake value.

## Consent

Written informed consent was obtained from the patient for publication of this case report and any accompanying images. A copy of the written consent is available for review by the Editor-in-Chief of this journal.

## Competing interests

The authors declare that they have no competing interests.

## Authors' contributions

IG was a major contributor to the manuscript. OR and AF performed the histological examination of the lung. FA, JS and EM performed the bronchoscopy and the follow-up of the patients. All authors contributed to the writing the manuscript and approved the final version.
